# Congenital Pulmonary Airway Malformation in an Adult Male Presenting With Hemoptysis

**DOI:** 10.7759/cureus.20862

**Published:** 2022-01-01

**Authors:** Marcos Valentin, Radhika Sharma, Jorge Trabanco, Tracy Ashby

**Affiliations:** 1 Internal Medicine, University of Florida College of Medicine – Jacksonville, Jacksonville, USA; 2 Pulmonary and Critical Care, University of Florida College of Medicine – Jacksonville, Jacksonville, USA

**Keywords:** bronchoscopy, minimally invasive lung resection, congenital pulmonary airway malformation, cystic lung disease, hemoptysis

## Abstract

Congenital pulmonary airway malformations (CPAMs) are rarely encountered in the adult population. Although they are typically diagnosed in the prenatal period, some may not cause symptoms and go unnoticed until adulthood. Patients with CPAM are at risk of developing pneumonia, hemorrhage, pneumothorax, and malignancy. There is a paucity of evidence regarding the management and prognostication of adults with CPAM. Patients often need to undergo surgical resection to prevent further episodes of infection, bleeding, or malignant transformation. Here, we present the case of an adult male with a CPAM who presented with frank hemoptysis. Computed tomography scan and bronchoscopy localized the lesion to the lingula. The patient underwent elective surgical resection of the lesion by video-assisted thoracoscopy and did not suffer any adverse outcomes. Surgical resection is generally recommended and appears to be a safe and effective approach to treating patients with symptomatic CPAMs. Inhaled tranexamic acid and bronchial artery embolization are valuable interventions in our armamentarium for managing hemoptysis but should not replace a definite surgical intervention due to the risk of recurrence.

## Introduction

Congenital pulmonary airway malformation (CPAM) is a rare congenital anomaly involving the lower respiratory tract. It is characterized by the development of cystic lesions which can arise anywhere along the bronchial tree or pulmonary acini. Although CPAMs are typically diagnosed perinatally by ultrasound or during infancy, sometimes they may remain undiscovered until adulthood. The severity of the disease can range from being asymptomatic to the development of severe hydrops fetalis in the prenatal period [1]. Asymptomatic patients who go on into adulthood may be discovered incidentally on plain chest radiography, but some may develop complications such as infection, pneumothorax, hemoptysis, or malignancy. Here, we describe a case of CPAM in an adult male presenting with frank hemoptysis who was treated with surgical resection.

## Case presentation

**Figure 1:**
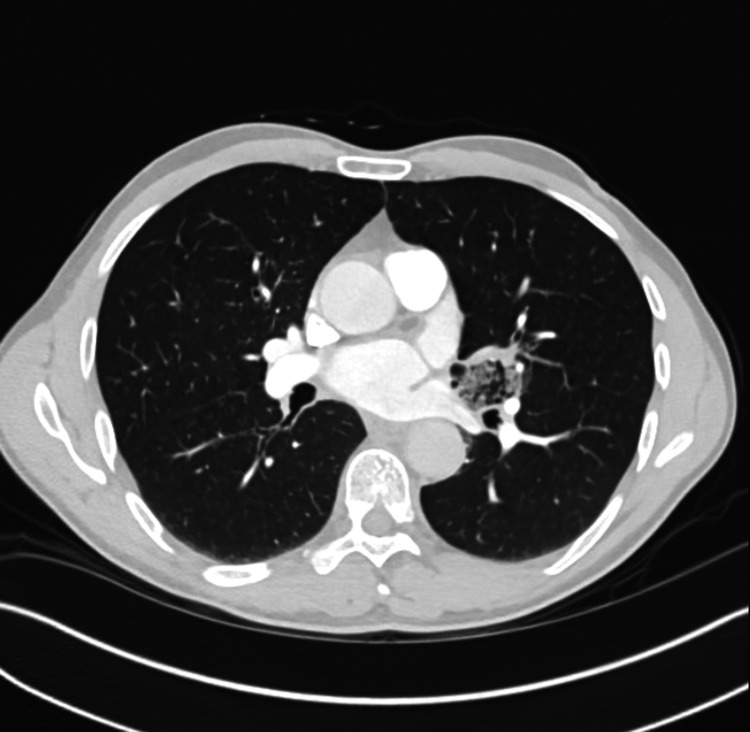
Computed tomography scan showing a left perihilar cystic lesion near the lingula.

A 50-year-old male from Egypt presented to the emergency department with sudden onset of frank hemoptysis. Prior to the presentation he had an estimated 200 mL of bloody sputum with visible clots and produced another 200 mL in the emergency room. He was not taking any blood thinners. A review of the systems was negative for chest pain, fevers, chills, night sweats, or weight loss. He had a cumulative 60-pack-year smoking history. Vital signs were within normal limits. His cardiopulmonary examination was notable for fine crackles and soft high-pitched wheezing localized to the left middle lobe. His complete blood count was within normal limits. A chest X-ray and computed tomography angiography were obtained (Figure 1) which revealed a lesion in the left perihilar region measuring 3.6 cm × 3.8 cm × 5.3 cm and comprising complex microcystic components. Inhaled tranexamic acid was used to relieve further episodes of hemoptysis. The patient underwent bronchoscopy with bronchoalveolar lavage which revealed active clots originating from the inferior segment of the lingula. He ultimately elected to undergo lingulectomy by video-assisted thoracoscopic surgery. The pathology showed dilated cystic airspaces and a focus of dilated vessels with variable wall thickening, hemorrhage, and reactive changes. The patient’s radiographic and pathology findings were consistent with a CPAM. He tolerated the procedure well and had an uneventful recovery period.

## Discussion

CPAMs are rare with an estimated incidence of 1 in 10,000 to 1 in 35,000 births [2]. The incidence in adults is even less frequent as they are usually discovered during prenatal screening by ultrasonography. There is limited evidence for managing CPAMS in adults which stems from case reviews and studies performed on the pediatric population [3-7]. This unique subset of patients is often asymptomatic and remains undiagnosed until later in life. They may be discovered incidentally on chest radiography or due to the development of symptoms, typically from lung infections.

There are five subtypes based on radiographic classification [1,8]. Type I is the most common and consists of one or more dominant cysts which measure 2-10 cm in size. Type II consists of smaller cysts <2 cm in diameter and is associated with other abnormalities such as congenital cardiac anomalies, renal agenesis, and pulmonary sequestration. Type III consists of microcysts that typically involve an entire lobe and has a poorer prognosis. Type IV is characterized by unlined cysts which typically affects a single lobe. Type 0 is lethal postnatally and is characterized by acinar dysgenesis or dysplasia. Other general imaging differentials to consider are bronchogenic cysts, pulmonary sequestration, congenital lobar emphysema, or congenital cystic bronchiectasis.

CPAMs can remain asymptomatic but can also pose a serious threat if left untreated. The most common complication of CPAMs is recurrent pneumonia. Other complications include the development of pneumothorax, hemoptysis, or malignant transformation. The incidence of malignant transformation is rare but is most commonly associated with type I and type IV malformations. Because the evidence for managing and prognosticating adults patients with CPAM is lacking, clinicians should invoke shared decision-making with patients. Asymptomatic CPAMs can be managed expectantly but elective surgical resection may also be offered on an individualized basis. A surgical approach is curative and often sought when complications such as infection or hemoptysis arise due to the risk of recurrence [9].

## Conclusions

In this case, the patient was diagnosed with a type III CPAM based on radiographic appearance. Our patient had a favorable outcome with early, non-emergent surgery. Based on this case, we urge clinicians to consider CPAM in patients who present for the first time with hemoptysis and abnormal chest imaging. In patients with severe or life-threatening hemoptysis, elective surgical resection is curative and should be performed due to the risk of recurrence. Perioperative use of inhaled tranexamic acid is helpful for relieving ongoing hemoptysis, and bronchial artery embolization may be a good option to treat acute bleeding causing hemodynamic or respiratory compromise; however, these interventions should be considered as a bridge and not a replacement to a definitive surgical intervention.
